# How to Make a Beetle Out of Wood: Multi-Elemental Stoichiometry of Wood Decay, Xylophagy and Fungivory

**DOI:** 10.1371/journal.pone.0115104

**Published:** 2014-12-23

**Authors:** Michał Filipiak, January Weiner

**Affiliations:** Institute of Environmental Sciences, Jagiellonian University, ul. Gronostajowa 7, 30-387 Kraków, Poland; University of Nebraska-Lincoln, United States of America

## Abstract

The majority of terrestrial biomass is wood, but the elemental composition of its potential consumers, xylophages, differs hugely from that of wood. This causes a severe nutritional imbalance. We studied the stoichiometric relationships of 11 elements (C, N, P, K, Ca, Mg, Fe, Zn, Mn, Cu, Na) in three species of pine-xylem-feeding insects, *Stictoleptura rubra*, *Arhopalus rusticus* (Coleoptera, Cerambycidae) and *Chalcophora mariana* (Coleoptera, Buprestidae), to elucidate their mechanisms of tissue growth and to match their life histories to their dietary constraints. These beetles do not differ from other Coleoptera in their absolute elemental compositions, which are approximately 1000 (N), 100 (P, Cu) and 50 (K, Na) times higher than in dead but undecayed pine wood. This discrepancy diminishes along the wood decay gradient, but the elemental concentrations remain higher by an order of magnitude in beetles than in highly decayed wood. Numerical simulation of the life history of *S. rubra* shows that feeding on nutrient-poor undecayed wood would extend its development time to implausible values, whereas feeding on highly decomposed wood (heavily infected with fungi) would barely balance its nutritional budget during the long development period of this species. The changes in stoichiometry indicate that the relative change in the nutrient levels in decaying wood cannot be attributed solely to carbon loss resulting from decomposer respiration: the action of fungi substantially enriches the decaying wood with nutritional elements imported from the outside of the system, making it a suitable food for wood-eating invertebrates.

## Introduction

The stoichiometric proportions of elements in plant and animal tissues differ substantially, which causes all herbivores to be confronted with a high “stoichiometric mismatch” [1], [2]: the proportions of C:x (C indicates the content of carbon, x indicates the content of another element) in the consumer's body are much lower than in its food. To build up and maintain its body composition, a herbivore must develop a strategy enabling the selective consumption or assimilation of particular elements. The strategies for how to cross such a stoichiometric threshold remain poorly understood. Invertebrates feeding on dead wood comprise an extreme case. C, H and O comprise 99% of wood mass. How can an organism build its body based on a substrate consisting almost exclusively of these elements? These compositional differences cause an extreme stoichiometric mismatch between the most common organic tissue on Earth and all the organisms exploiting this resource.

The problem of the stoichiometric mismatch between plant biomass and its potential non-microbial consumers in terrestrial ecosystems has not been fully investigated. Fagan et al. [3] observed the discrepancy in nitrogen and phosphorus content between living autotrophs (plant foliage and algae) and typical herbivores; the C:N and C:P ratios are 10 to 20 times higher in autotrophs than in herbivores. Schneider et al. [4] demonstrated a high stoichiometric mismatch in the P content between cave-dwelling invertebrates and the plant detritus they consume. Wood-boring (xylophagous) insects living in the xylem of dead trees constitute another prominent example but have not previously been studied. As estimated from the available data on the elemental content in wood [5]–[7] and insects [3], [4], [8], [9], the stoichiometric discrepancy may reach two orders of magnitude for living wood and three orders of magnitude for dead wood. This discrepancy concerns not only nitrogen and phosphorus but also K and Mg. Many of the studies on the nutritional mismatch between terrestrial herbivores and their food have focused exclusively on two major nutrients: N and P; however, other elements are also essential for the growth and survival of consumers, and a low level of these elements in food plants may cause a nutritional imbalance [9]–[11]. No data are available for the content of minor essential nutrients in dead wood; however, it may be assumed that their concentrations are also negligible. To date, only a few papers have tackled the stoichiometry of microelements, including studies of freshwater or marine pelagic systems [12]–[14], of terrestrial saprobiotic microorganisms [15], and of prairie grasshoppers [10]. However, the majority of those studies concerned the consumption of living plant tissues, which are relatively nutrient-rich [3], [10], [16]. Bark beetles, feeding on living phloem, are the most unbalanced herbivores studied to date [2], [8], [16], [17]. Sterner and Elser [16] suggested that termites may possibly be the most unbalanced consumers, with a discrepancy of two orders of magnitude between the nutrient content of their bodies and the nutrient content of their food.

Stoichiometric mismatch is not the only problem faced by xylophagous organisms. The poor digestibility of cellulose, hemicelluloses and lignin limits the energy budgets of wood-eating invertebrates and slows their growth [18]–[20]. The life strategy of xylophages includes an extremely long development time, which is possible due to the relatively low mortality risk of larvae living deep in the xylem and may compensate for the low digestibility of food [20], [21]. However, it remains an open question whether the fundamental effect on the life history of a xylophage is caused by carbon (energy source) or other nutrients (assembling molecules of crucial functional importance). Although the symbiotic interactions of numerous xylophages with microorganisms may ease the cellulose digestibility constraint [22], the supply of nutrients in wood cannot be increased this way; their deficit can only be ameliorated by an external supply. However, despite the stoichiometric mismatch, wood-boring beetle larvae are capable of extracting from their food all of the elements necessary for growth and for controlling their metabolic processes. Therefore, the wood consumed must be supplemented with some nutrient-rich material. The obvious candidates are saprobiotic fungi that invade dead wood, which are capable of transferring large quantities of elements to the food source (see [23] for a review).

The aim of this study was to determine how xylophagous insects can manage the drastic stoichiometric imbalance of major and minor nutrients. We tested the hypothesis that rather than offsetting nutritional constraints with a prolonged development period, wood-boring larvae may balance their nutritional demands by the import of nutritional elements from outside the system by the action of fungi. Thus, avoiding the stoichiometric mismatch may differentially shape the life histories of dimorphic sexes and various species exploiting the same resources. To address this hypothesis, we compared the elemental contents in the bodies of three species of wood-boring beetles inhabiting the same pine stumps, differing in body size and life histories, with the elemental content of wood (potential food during larval development) at various stages of decay. We examined the levels of essential macro- and micronutrients (C, N, P, K, Ca, Mg, Fe, Zn, Mn, Cu, and Na). In decaying wood, in addition to elemental concentrations, we also estimated the amount of fungal tissue using ergosterol content as a proxy [24].

## Materials and Methods

Three common species of pine-xylem-feeding beetles were used: *Stictoleptura rubra* Linnaeus 1758 ( =  *Corymbia rubra* Nakano and Obayashi 1957;  =  *Aredolpona rubra* Viliers 1974), *Arhopalus rusticus* Linnaeus 1758 ( =  *Criocephalus rusticus* Haldeman 1847; Coleoptera, Cerambycidae) and *Chalcophora mariana* Linnaeus 1758 ( =  *Buprestis mariana* Linnaeus 1758; Coleoptera, Buprestidae). The development times for these species reported in the literature [25] are 3 years in the smallest beetle, *S. rubra*; 2–4 years in *A. rusticus*, which is of intermediate size; and 5–6 years in the largest of these beetles, *Ch. mariana*. Pine stumps potentially inhabited by larvae of these beetles were collected in the Niepołomice Forest, approximately 20 km east of Cracow (southern Poland, 50°05′N, 20°21′E, elevation 184–212 m a.s.l.) in spring, summer and autumn during 2010–2012. The stumps were collected from approximately 80-year-old pine stands, one to four years after felling. The stumps (diameter measured at the top was approximately 40 to 90 cm; height measured at the center was approximately 10 to 50 cm) were cut slightly below the ground and were hand-split to collect wood samples, pupae and larvae. The adult beetles leaving the stored stumps were also collected. In addition, *S. rubra* adults were captured in the forest. The field studies did not involve endangered or protected species. No specific permissions were required for collecting beetles and stumps in this location.

The wood samples were classified by degree of decay (after [26]): (1) undecayed wood – hard and healthy, without visible changes caused by microorganisms; (2) moderately decayed wood – considerably changed by microorganisms, colored (purple or dark brown), wet and softer than (1) but still difficult to tear apart with a knife; (3) highly decayed wood – many visible changes, ample layers of white or brown rotting fungi, wet and soft, easily torn apart by knife or even by hand; and (4) corridor wood – a thin layer of wood from the walls of corridors made by xylophagous larvae, together with content (‘frass’, i.e., wood fragments and feces).

Prior to elemental content determination, the insects and wood samples were freeze-dried (using Christ Beta 1–8 LD plus). All the samples were dried using a two-stage pressure/temperature regime: main drying at 0.34 mbar/−31°C and final drying at 0.0010 mbar/−76°C. We dried insects for five days and wood samples for seven days. For CHNS analyzer, we dried, ground and homogenized the material (one insect or wood sample taken from one stump per measurement sample), and for the mineralization (samples for AAS and colorimeter) we dried intact insects and ground and homogenized wood (wood sample taken from one stump per measurement sample). C and N levels were determined using a Vario EL III automatic CHNS analyzer. K, Ca, Mg, Fe, Zn, Mn, Cu and Na levels were determined by atomic absorption spectrometry (Perkin-Elmer AAnalyst 200 and Perkin-Elmer AAnalyst 800), and P content was determined colorimetrically (MLE FIA flow injection analyzer). Prior to analysis, the samples were mineralized by acid digestion: beetles in HNO_3_, pupae in a solution of HNO_3_, HClO_4_ and H_2_SO_4_, and wood samples in a solution of HNO_3_ and HClO_4_. Insect samples consisted of one or a few individuals (as described above), and pupae, adult males and adult females were considered separately. Sulfanilic acid was used as the reference material for C and N analyses, and Certified Reference Materials (bush – NCS DC 733348, chicken – NCS ZC73016 and pork muscle – NCS ZC 81001) were used for the other elements. Sample sizes differed for particular elemental analyses of beetles and variously decayed wood because of the uneven abundance of particular species, sexes and developmental stages and the necessity to pool small specimens and match the separate requirements for every individual element measured (for details, see S1 Table).

Ergosterol content was measured in wood samples using a GC/MS Clarus 600 chromatograph (Perkin-Elmer). We used 73 wood samples collected from the same site as the samples for the elemental content analysis, but from different stumps. The degree of decay of the stumps was categorized in the same way as for elemental content analysis, but the 4th category (corridors) was omitted.

We express the degree of stoichiometric mismatch between the insects and their food for element x most simply as the ratio of the stoichiometric ratios in food and in the consumer's body (Trophic Stoichiometric Ratio  =  *TSR*):
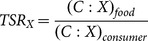
(1)where *C* – carbon content and *X* – content of element *x*.

This index does not depend on the units used for stoichiometric ratios C:x (molar or mass units). Values of *TSR_x_>1* indicate stoichiometric mismatch, with severe mismatch indicated by a *TSR* value substantially different from unity.

Because the variation of the *TSR* values cannot be directly measured, we used bootstrapping to estimate the mean *TSRs* for specific elements and food categories with confidence limits. First, the *TSR* values were computed from raw data on a given elemental content in the food and consumer. The values were drawn 15 times, and a mean was calculated. The procedure was repeated 500 times, giving 500 means (bootstrap samples), from which the final averages with confidence intervals were calculated.

Principal component analysis (PCA) was employed to compare the multi-elemental stoichiometric relations among species, sexes and developmental stages. The data were log-transformed, centered and standardized by species but not by sample; thus, PCA was performed on a correlation matrix. To check for differences between the indicated clusters, we computed ANOVA independently for the 1st and 2nd axis scores.

The Mann-Whitney U test and Kruskal-Wallis test were used for significance testing (p<0.05) of the differences between species and sexes in the elemental composition and body size (dry mass). Statistica 10 was used for all statistical analyses except for PCA (Canoco 4.5).

## Results

### Body composition of beetles

**Figure 1:**
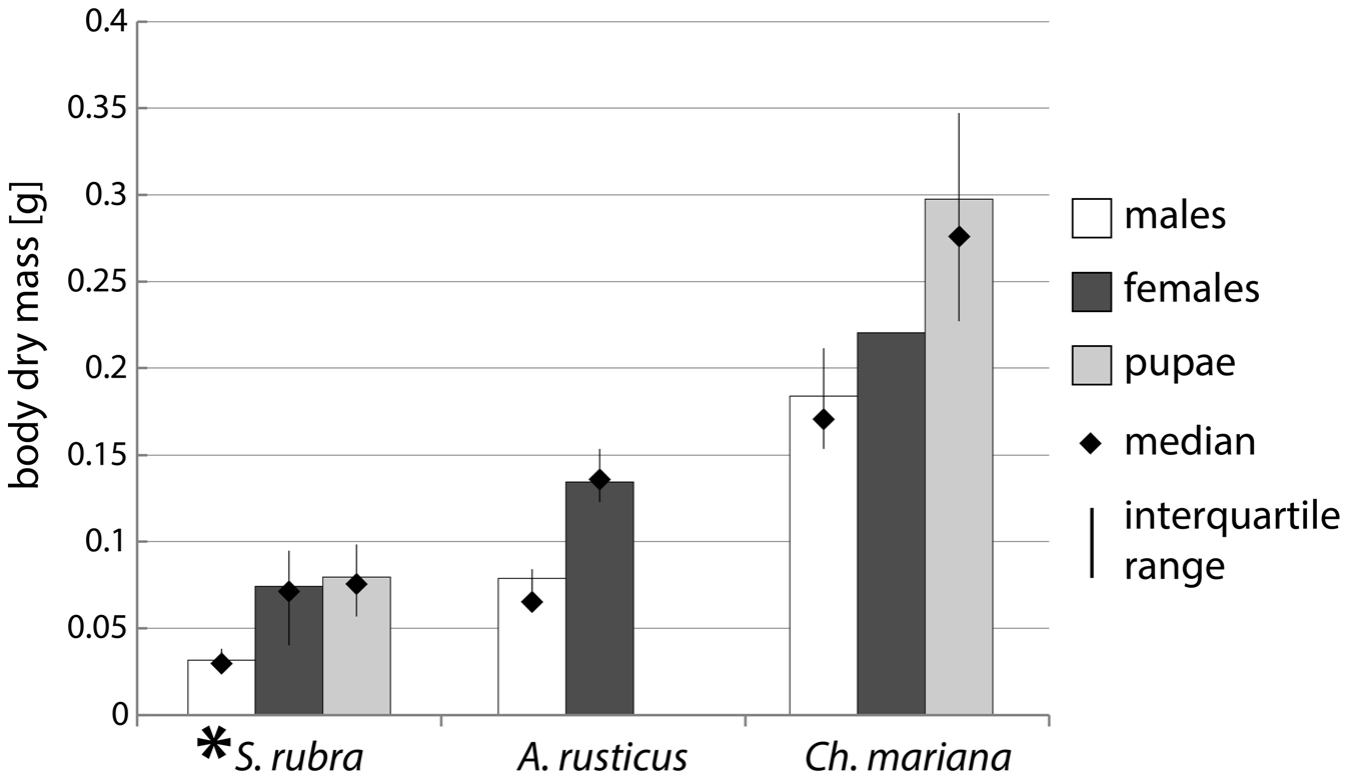
Body sizes of the studied xylophagous beetles (dry mass, boxes  =  means). Significant differences between males and females are asterisked (Mann-Whitney U test, p<0.05). *Ch. mariana* was not considered in significance testing (too few female specimens).

The adult beetles differed in body dry mass between species and showed sexual dimorphism of body size, with females being larger than males (Fig. 1; for details, see S2 Table), but the difference in body size between sexes was significant only for *S. rubra* (Mann-Whitney U test, p<0.05).

The sexes of the pupae were not determined; their mass was intermediate between the adult masses of the two sexes or larger than the masses of either (Fig. 1; for details, see S2 Table).

The complete data on elemental content are presented in S1 Table. The carbon mean content in imagines ranged from 51.7 to 63.3% d.m. and did not differ significantly between species or sexes, except for *A. rusticus* females, which had a significantly higher carbon content than males and females of other species. *Ch. mariana* pupae had a significantly lower C content than *S. rubra* pupae (Table 1).

**Table 1:** Average concentrations of elements in the three species of xylophagous beetles and in samples of variously decayed wood from pine stumps inhabited by larvae (see text for definitions of wood decay categories).

			C	N	P	K	Na	Ca	Mg	Fe	Zn	Mn	Cu
Xylophages			% d.m.			**mg/kg d.m.**							
	*S. rubra*	mean	52.9	10.3	0.6	6866.8	711.6	770.3	1428.8	102.9	112.8	23.8	37.2
		SD	3	1.3	0.1	931.8	259.7	433.2	266.8	41.6	18.6	5.4	18.6
	sex diff.		n.s.	M>F	n.s.	n.s.	n.s.	M<F	M<F	n.s.	n.s.	n.s.	n.s.
		mean	59.2	7.8	0.6	6831	794.1	1421.4	1615.9	128.3	175.9	18.8	41.6
	*A. rusticus*	SD	5.1	2.2	0.1	1081.8	207.8	1124.3	284.3	69.2	67.3	6.5	10.9
	sex diff.		M<F	M>F	M>F	n.s.	n.s.	M>F	n.s.	M>F	n.s.	n.s.	n.s.
		mean	54.6	7.9	0.6	7837.1	818.1	808	2202.6	42	78	25.3	6.8
	*Ch. mariana*	SD	1.8	1	0.1	890.1	499.4	136.6	441.9	10.8	12.1	6.3	3
	sex diff.		n.s.	M>F	n.s.	n.s.	n.s.	n.s.	n.s.	n.s.	n.s.	n.s.	n.s.
	Pooled	mean	54.1	9.4	0.6	7137.9	752.2	879.4	1684.3	80	109.6	23.6	28.7
		SD	3.7	1.8	0.1	1027.7	339	556.7	476.3	51.8	44.4	6.2	20.5
	males species diff.		n.s.	Sr>Cm	n.s.	n.s.	Sr<Cm	Sr<Ar	Sr<Cm	Sr = Ar>Cm	Sr = Ar>Cm	n.s.	Sr = Ar>Cm
	females species diff.		Sr = Cm<Ar	Sr = Cm>Ar	n.s.	n.s.	n.s.	n.s.	n.s.	n.s.	n.s.	n.s.	n.s.
	pupae species diff.		Sr>Cm	Sr>Cm	Sr<Cm	Sr<Cm	n.s.	n.s.	Sr<Cm	Sr>Cm	Sr>Cm	n.s.	Sr>Cm
			% d.m.	**mg/kg d.m.**									
Wood	Undecayed	mean	55	84.7	10.1	147.7	16.4	986.1	116.3	13.9	9.9	57.7	0.4
		SD	2.8	53.1	6.2	47.5	2.2	230.2	53	1.4	3.5	28.9	0.1
	Moderately decayed	mean	50.8	370	54	274.7	15.5	1027.3	122.1	29.2	10.8	48.9	3
		SD	1.6	177.6	21.3	121.4	5.7	314.2	60.2	13.2	4	13.9	2.6
	Highly decayed	mean	48.9	2166.5	149.6	735.2	22.3	1301.4	196.3	32.8	11.9	68	2.9
		SD	0.7	863.5	74.7	371.4	6	323	81.7	18.3	2	22.1	1.8
	Corridors	mean	49.6	1178.3	102	744.8	41.4	1187	296.3	30.1	17.1	62	2.5
		SD	1.2	687	46	423.6	17.8	497.6	177.4	15.7	5.2	23.2	1.7
	wood categories diff.		1 = 2>3; 4<1	1 = 2<3; 4>1	1 = 2<3 = 4	1 = 2<3 = 4	1 = 2 = 3<4	n.s.	4>1 = 2	1<2 = 3 = 4	1 = 2 = 3<4	n.s.	1<2 = 3 = 4

sex diff. – significant differences between sex categories (Mann-Whitney U, p<0.05), M – male, F – female; species diff. – significant differences between species of one sex/age category (males, females, pupae) (Kruskal-Wallis test or Mann-Whitney U, p<0.05); Sr – *Stictoleptura rubra*, Cm – *Chalcophora mariana*, Ar – *Arhopalus rusticus*; wood categories diff. – significant differences between wood decay categories (Kruskal-Wallis test, p<0.05).

The mean nitrogen content ranged from 6% to 10.9% and differed significantly between species and sexes. The N content was significantly lower in females of all the species, and the pupae showed the lowest values (Table 1).

Males of all three species had similar P levels (av. 0.64%, SD 0.1; Table 1). Male and female *S. rubra* did not differ in P content, but their pupae had a significantly lower P content than adults. The P content differed significantly between the sexes in *A. rusticus*, and the pupae of *Ch. mariana* had a significantly higher P content than *S. rubra* pupae (Table 1).

The levels of the eight other elements did not differ significantly within species, with only three exceptions: Ca (lower in male than in female *S. rubra*, higher in male than in female *A. rusticus*, Mann-Whitney U test, p<0.05; Table 1), Mg (lower in male than in female *S. rubra*; Table 1), and Fe (higher in male than in female *A. rusticus*; Table 1). The interspecific differences appear to reflect taxonomic relationships at the level of the subfamilies Buprestidae and Cerambycidae (Table 1): *Ch. mariana* pupae had higher K and Mg contents and lower Fe, Zn and Cu contents than *S. rubra* pupae. *Ch. mariana* males had higher Na and Mg contents and lower Fe, Zn, ad Cu contents than *S. rubra* males. *A. rusticus* males had higher Ca content than *S. rubra* males.

**Figure 2:**
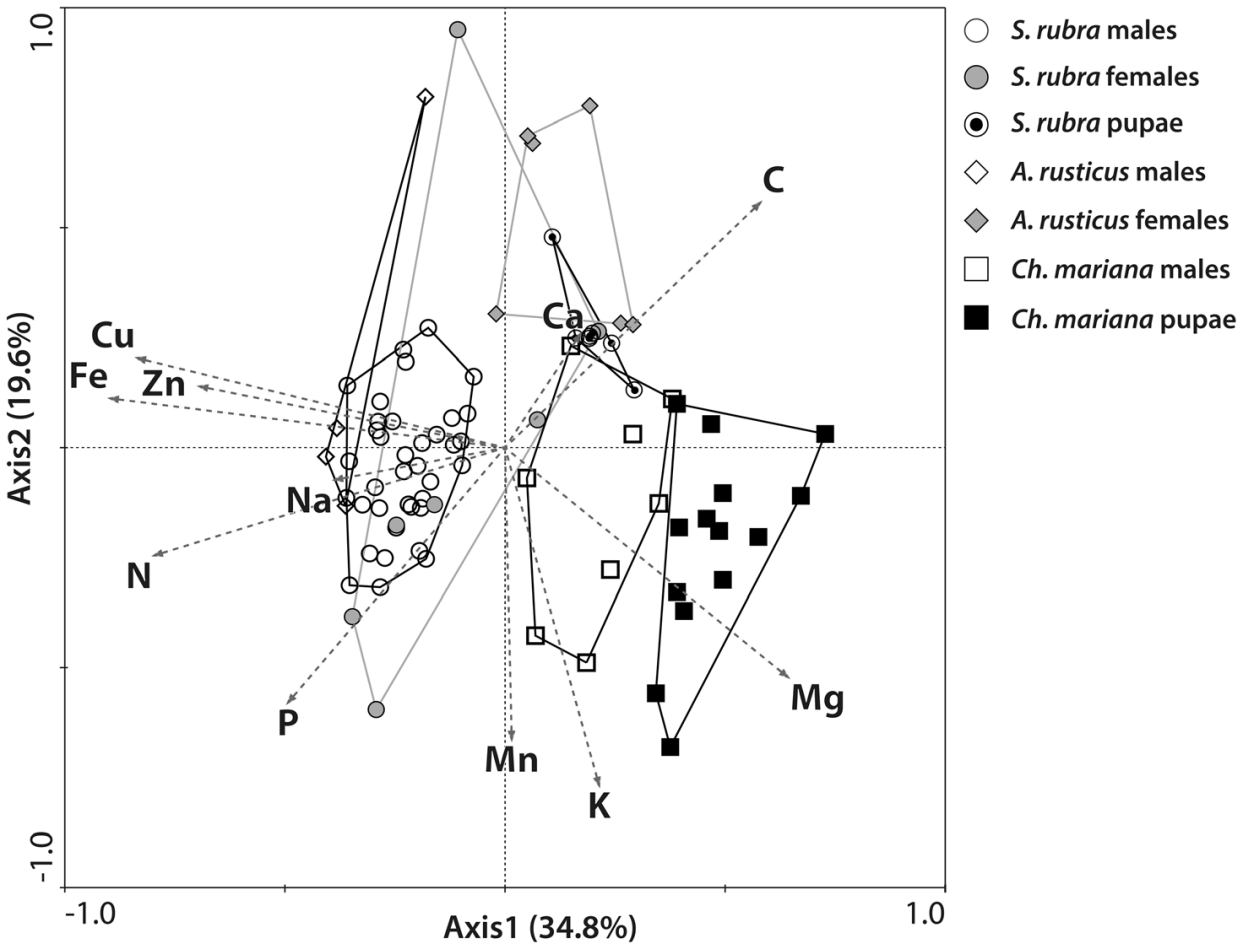
Multivariate analysis of stoichiometric relations in three species of xylophagous beetles based on the 11 studied elements – PCA plot (first two axes).

**Figure 3:**
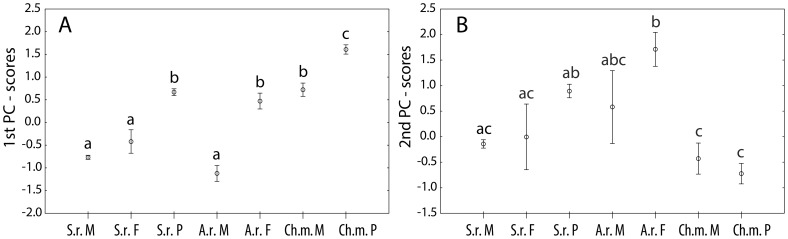
Multivariate analysis of stoichiometric relations in three species of xylophagous beetles (PCA). ANOVA computed independently for the 1st and 2nd axis scores A – ANOVA for scores of the 1st principal component (F6, 76 = 91.919, p = 0.0000); B – ANOVA for scores of the 2nd principal component (F6, 76 = 8.8204, p = 0.00000); vertical bars denote standard errors. Values bearing different letters denote significant differences in the elemental composition between species, sexes and pupae (unequal N, HSD test, p<0.05). S.r. M - *Stictoleptura rubra* males, S.r. F - *Stictoleptura rubra* females, S.r. P - *Stictoleptura rubra* pupae, A.r. M - *Arhopalus rusticus* males, A.r. F - *Arhopalus rusticus* females, Ch.m. M - *Chalcophora mariana* males, Ch.m. P - *Chalcophora mariana* pupae.

PCA allowed for a simultaneous comparison of multi-elemental stoichiometries in all sex, age and species categories of the beetles studied. On the plane determined by the first two axes (54.4% of the total variance), the beetles tended to group according to taxonomy (species and subfamily) and sex (Fig. 2). The 1st component was loaded mostly by the variance of Fe, Cu, N, and Mg, and the 2nd component was loaded by K, Mn and P levels. The clusters for both sexes of *S. rubra* greatly overlapped but differed from the pupae of this species (Fig. 2). The other two species tended to differ between themselves and to maintain the body multi-elemental stoichiometry across developmental stages and across sexes; no overlap occurred between clusters for *Ch. mariana* pupae, *Ch. mariana* males, and the clusters for the representatives of Cerambycidae (Fig. 2). These tendencies were partly confirmed as statistically significant by the ANOVA computed independently for the 1st and 2nd axis scores (Fig. 3).

### Elemental content and ergosterol content in wood

**Figure 4:**
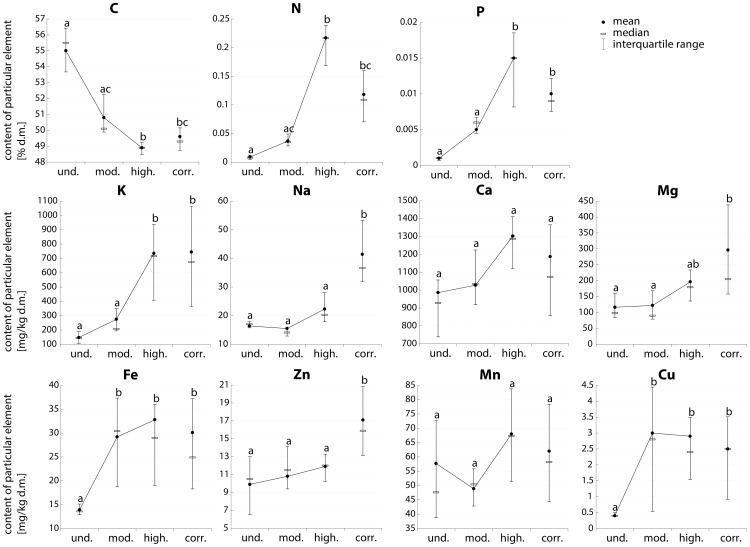
Decay-related changes of elemental content in pine stumps inhabited by larvae. und. - undecayed wood; mod. - moderately decayed wood; high. - highly decayed wood; corr. - corridors (see text for definitions of wood decay categories). Values bearing the same letter do not differ significantly between wood decay categories (Kruskal-Wallis test, p<0.05). For detailed results, see S1 Table.

The relative concentrations of all elements except carbon tended to increase during the decay process. In the material from corridors, the elemental concentrations were similar to those in highly decayed wood, with the exception of Na, Zn and Mg (concentrations highest in corridors; Fig. 4, see S1 Table for complete data set). The relative increments of the increase in concentration during wood decay (from category 1 to 3) were highest for nitrogen (23-fold), phosphorus (14-fold), copper (6.3-fold) and potassium (4-fold); the other elemental concentrations increased by 18% (Mn) to 136% (Fe).

**Figure 5:**
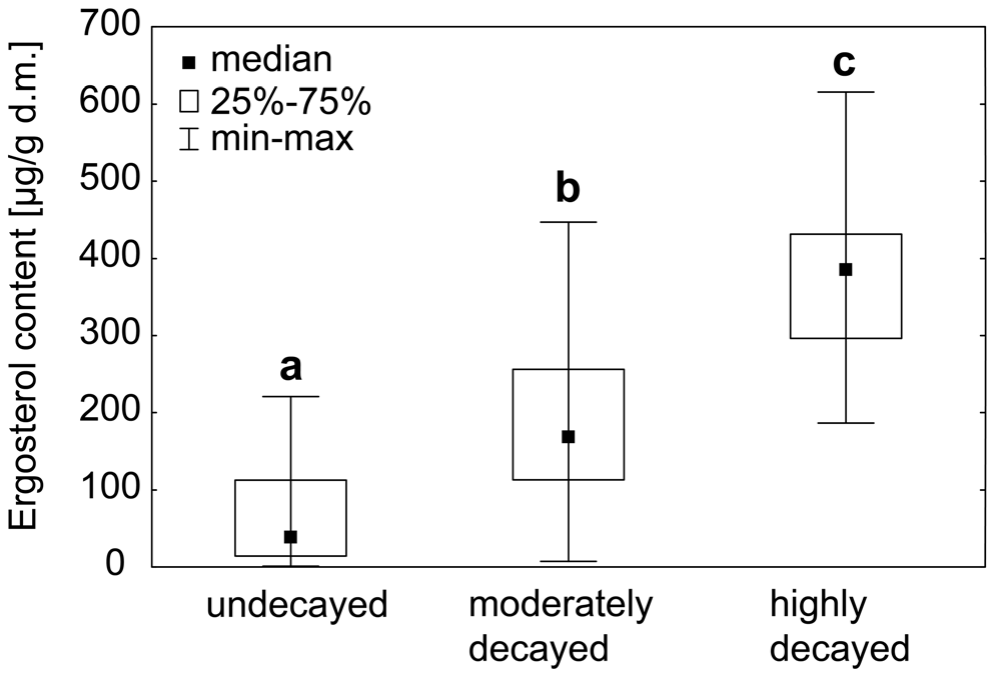
Changes in the concentration of ergosterol in dead wood along the decay gradient. Boxes bearing different letters denote significant differences in ergosterol content (Kruskal-Wallis test, p<0.05).

Ergosterol content in dead wood significantly increased along the decay gradient; each wood category significantly differed from the two others (Kruskal-Wallis test, p<0.05, Fig. 5). The respective median values were 39.6 µg/g (dry mass) for the undecayed wood, 169 µg/g for the moderately decayed wood and 385.7 µg/g for the highly decayed wood (see S4 Table for more details).

### Stoichiometric mismatch expressed as the Trophic Stoichiometric Ratio (TSR values)

The *TSR* values were highest for the undecayed wood for all the nutrients (Table 2, S3 Table). After pooling the data for the three species and both sexes (Table 2), the stoichiometric mismatch of undecayed wood as food, represented by the *TSR* values, reached three orders of magnitude for N, two orders of magnitude for P, one order of magnitude for K, Na, Mg, Zn and Cu, and less than one order of magnitude for Fe. No stoichiometric mismatch was shown for Ca and Mn: the *TSR* values for these nutrients were lower than one. The stoichiometric mismatch was lower in moderately decayed wood (two orders of magnitude for N and P, one order of magnitude for K, Na, Mg, Zn and Cu) and even lower for highly decayed wood (one order of magnitude for N, P, K, Na, Zn and Cu). The *TSR* values calculated for corridors were between those for moderately decayed wood and highly decayed wood for most of the elements but were the lowest for Na, Mg and Zn (Table 2).

**Table 2:** Trophic stoichiometric ratios (*TSR  =  (C:x)_wood_/(C:x)_beetle_*); averages (lower bound – upper bound) of the bootstrapped distributions.

Wood decay categories	Elements									
	N	P	K	Na	*Ca*	Mg	Fe	Zn	*Mn*	Cu
Undecayed	**2246**	**861**	**54**	**50**	*0.99*	18	7.4	14	*0.48*	**86**
	**(1306–3327)**	**(681–1088)**	**(46–63)**	**(42–61)**	*(0.72–1.33)*	(13–23)	(5.6–9.5)	(11–18)	*(0.38–0.58)*	**(63–112)**
Moderately decayed	**364**	**139**	**28**	**51**	*0.93*	14	4	13	*0.47*	**32**
	**(203–656)**	**(92–199)**	**(22–34)**	**(36–68)**	*(0.52–1.43)*	(10–19)	(2.3–6.2)	(8–21)	*(0.34–0.62)*	**(11–60)**
Highly decayed	**50**	**49**	**12**	**34**	*0.69*	9	3.4	10	*0.33*	**15**
	**(40–61)**	**(39–62)**	**(9–14)**	**(27–42)**	*(0.50–0.94)*	(7–11)	(2.5–4.5)	(8–12)	*(0.27–0.40)*	**(10–22)**
Corridors	**128**	**87**	**13**	**23**	*0.85*	7	3.8	7	*0.39*	**25**
	**(77–189)**	**(55–146)**	**(9–16)**	**(16–33)**	*(0.58–1.21)*	(5–9)	(2.7–5.0	(6–9)	*(0.31–0.48)*	**(14–38)**

**Bolded letters** indicate the most limiting nutrients (*TSR>10* for all the wood categories); *italics* – nutrients in excess (*TSR <1* for all the wood categories); see text for definitions of wood decay categories; pooled data for 3 species, both sexes (number of specimens as in Table 1); see text for explanation of bootstrap calculation and S3 Table for detailed results.

## Discussion

Our results show that dead wood tissue as the sole source for xylophagous beetles cannot provide sufficient nutrients for growing larvae to compose their bodies. In addition to N and P, we found that K, Na, Mg, Fe, Zn and Cu are also limiting nutrients. The larval diet is apparently supplemented by fungal tissues gradually infecting the decaying wood and transporting nutritional elements into the stump. Thus, “xylophages” (also called “wood eaters”) are in fact “fungivores”, as they feed on fungi. The nutritional demands differ among species belonging to different families, as determined by the content of several elements in the adult bodies: Fe, Zn, Cu, Mg, Na, and N. Within a family, the species do not differ significantly in elemental composition, but the sexes do differ in their elemental composition. The females require more time to develop because they are larger. The pine stumps in this study were inhabited mostly by *S. rubra* and *Ch. mariana*, which belong to different families and differ significantly in their body stoichiometries and life histories. However, the specimens of *A. rusticus*, a species quite similar to *S. rubra* in stoichiometry and in the length of development time, only rarely co-occurred in the stumps.

### Stoichiometry of xylophages

For the three major body components (C, N, P), the body composition of adult xylophagous beetles falls within the broad range of values found in the few other coleopteran taxa studied to date [3], [8], [27], [28]. It has been suggested that besides a taxonomic idiosyncrasy or a presumed body mass allometry, N and P content may reflect the feeding strategy of invertebrates, with predators having higher concentrations of N and P than herbivores and detritivores [3], [27]. The xylophagous beetles studied here do not confirm this generalization: their N content is close to the high values reported for carnivores, and their P content is intermediate between herbivores and carnivores [3], [27]. However, our results are consistent with species-specific data provided by Fagan et al. [3], who found high N levels in several cerambycid and buprestid beetles. Females of all three species studied here have significantly lower N concentrations than males of the same species, possibly associated with the higher fat content in the females.

Because the sex of pupae could not be determined, and the samples most likely contain individuals of both sexes, their average body composition should be intermediate between males and females. The position of pupae of the two species studied on the PCA plot (Fig. 3) suggests that they have relatively high C levels and low N and P levels. This result may be attributed to the higher C:N ratio of the chitinous exuvium left behind at eclosion and the fat reserves exhausted during the pupal stage.

### Stoichiometry of wood decay

The concentrations of the nutrients (N, P, K, Ca, Mg) measured in the sapwood and heartwood of living gymnosperm trees [5] are an order of magnitude greater than in the undecayed wood of the dead pine stumps that we studied. The stumps, cut one to four years before the wood samples were taken for analysis, were remnants of trees cut when they were at least 80 years old. Palviainen et al. [6], [7] reported similarly low nutrient concentrations measured in pine stumps during the first five years of decay after cutting. Meerts [5] suggested that mineral nutrients in a living tree may be recycled from senescing sapwood. After cutting, the stumps are exposed to weathering and may be further depleted of nutrients until the fungal mycelium growing into the wood enriches it with nutrients, particularly N and P, imported from outside the system [29]–[31]. Even so, there is still a significant difference in the stoichiometries between partly decayed wood and the insects feeding on it. The nutritional composition of corridor material is similar to that of the wood from which the samples were taken (moderately and highly decayed), except for Na, Zn and Mg, which are present in the corridors at higher concentrations (Fig. 4). Most of the corridors that we measured originated from moderately decayed wood and less commonly from highly decayed wood. It is not clear whether the chemical composition of the wood is nonuniform and the larvae bore selectively in the more nutritious areas or whether the chemical composition of the corridors changed due to the more intense penetration of fungal mycelium following larval activity.

### The dynamics of nutrient content in decaying wood

Two mechanisms contribute to the decrease of C:x ratios during decay, that is, to the enrichment of the wood with elements other than C, H and O: (i) the liberation of C as CO_2_ due to microbial and animal respiration, and (ii) the import of nutrients from outside the system by fungal tissue (mycelium) growing into the decaying wood. To determine whether elements are imported in significant amounts during wood decay, we assessed the relative contributions of the two mechanisms from the stoichiometric proportions of specific elements in the wood at various stages of decay.

The initial (*SRI*) and final (*SRF*) stoichiometric ratios in decaying wood for a given element x may be expressed as:
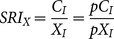
(2)

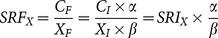
(3)where *pC_I_* and *pX_I_* are initial relative concentrations of carbon and of element *x*, respectively; *C_I_*, *C_F_*, *X_I_* and *X_F_* are the initial and final absolute amounts of carbon and of element x, respectively; α represents the proportion of carbon not released as CO_2_ during decay; and *β* represents the coefficient of enrichment of element *x*.

From equation (3), it follows that 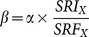
(4)


A value of *β>>1* would indicate an increase of the absolute amount of element *x* in the wood during decay. The coefficient *α* is defined as:
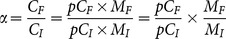
(5)where *M_I_* and *M_F_* are initial and final masses of decaying wood, respectively.

The concentration of carbon decreases from 55% to 48.9% during decay (Table 1); thus, *pC_F_/pC_I_  = 0.489/0.550 = 0.889*. The unknown proportion *M_F_/M_I_* can approximated from literature data concerning the rates of decay of coarse woody debris (e.g. [32], [33]), including those of conifer stumps [34], [35]. The proportion of the remaining mass of wood *d* after *t* years of decomposition is usually described with exponential model 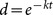. The experimentally evaluated constant *k* may range between 0.020 and 0.1101, depending on the tree taxon, environmental conditions and the methods used [32]–[35]. Samples of wood at advanced stages of decay were taken from the stumps of trees cut 3 or 4 years before sampling. The model solved for 3 years of decay using these figures yields the proportion of remaining mass between 0.72 and 0.94 and solved for 4 years 0.64 to 0.92. Based on these estimates, the possible values of the coefficient α (eq. 5) may range from 0.57 to 0.84. The range of *β* values can be estimated for each element *X* using *β_x_ = α×SRI_x_/SRF_x_*, where *SRI_x_* and *SRF_x_* are the measured initial and final *C/X* ratios. The results (Table 3) show that independently of the assumed value of α, the amounts of Na, Ca, Mg, Zn, Mn and Fe do not increase during decay (coefficients of enrichment do not deviate substantially from 1.0, ranging from 0.7 to 1.4 for *α* = 0.57 and from 1.0 to 2.1 for *α* = 0.84), but the contents of N, P, Cu and K increase several fold, independent of the assumed value of *α* (Table 3). Thus, the change in the relative concentrations of these elements is not only the result of the carbon escape from decaying wood but also the result of the net import of these elements from outside of the system.

**Table 3:** Coefficient of enrichment of elements in decaying wood (see text for explanation).

			N	P	K	Na	Ca	Mg	Fe	Zn	Mn	Cu
C:x in undecayed wood		*SRI*	6111	55000	0.4	3.4	0.1	0.5	4	5.6	1	137.5
C:x in highly decayed wood		*SRF*	253.5	3667	0.1	2.5	0.04	0.3	1.7	4.6	0.8	19
		*α = 0.84*	**20.1**	**12.5**	**3.3**	1.1	**2.1**	1.4	2	1	1	**6**
Coefficient of enrichment	*β*	*α = 0.57*	**13.7**	**8.5**	**2.3**	0.8	1.4	0.9	1.3	0.7	0.7	**4.1**

Bolded letters indicate the coefficients of enrichment exceeding 2, indicative for the elements imported to the decaying wood from outside.

Although part of the additional N may be supplied by nitrogen-fixing bacteria, which are known to occur in decayed plant matter [36], fungi appear to be the only agents translocating other nutrients from outside of the system to the decaying wood. In fact, the ergosterol content (the fungal tissue proxy) increased along the decay gradient and differed significantly between dead wood categories (Fig. 5). The measured ergosterol contents cannot be simply recalculated into fungal biomass because the conversion factors are strongly dependent on the fungal species [37]. However, the contents of N, P. K and Cu in the mycelium of wood decaying fungi [38], [39] and in the fruiting bodies of mushrooms [38], [40] are two to three orders of magnitude higher than in undecayed wood (this study), while the contents of the other elements studied here differ less than one order of magnitude.

Thus, the increase in nutrient concentration in decaying wood may be attributed to the action of fungi. An experiment in which xylophagous beetle larvae were fed with fungi instead of wood [41] showed that fungi can be an adequate food source for these insects. Fungi inhabiting dead wood have been described as nutrient immobilizers [42], and our data support the view that fungi may serve as nutrient deliverers [6], [7], [30], [31], [43], [44]. The translocation of elements by fungi in the forest floor is a well-known phenomenon [29], [30], [45], [46].

### Nutritional imbalance in wood-boring insects

The concentrations of elements in the imagines and pupae were one or more orders of magnitude greater than in the potential food of the larvae, except for Ca, Mn and Fe, which showed a less pronounced discrepancy (Table 1). The mismatch, expressed as the *TSR* value, is most striking for undecayed wood (three orders of magnitude for nitrogen) and diminishes as wood decay proceeds, but the differences remain large (Table 2; for full data set, see S3 Table). The nutrient content of wood from corridors is close to that of highly decayed wood (Table 2). The most extreme differences are for N and P, which are, respectively, 1500–2000 and 500–900 times less concentrated in undecayed wood than in the beetles. The concentration differences for Cu, K and Na are also significant. Cu is approximately 86 times higher in beetles than in undecayed wood. The K and Na concentrations are 54 and 50 times higher, respectively, in the beetles than in undecayed wood (Table 2). Only Ca and Mn are available in excess. The other nutrients (K, Na, Mg, Fe, Zn, Cu) are much more scarce in dead wood, such that they may constrain the development of wood-eating larvae. All the elemental concentrations tend to increase as wood decay proceeds (Table 2, Fig. 4), although the *TSR* values remain quite high for N and P. Considering wood as the exclusive source of nutrients, xylophagous beetles seem to be faced with the most unbalanced diet of all organisms studied to date. Even termites live on a less unbalanced diet [16].

The N and P stoichiometric mismatches were similar in all three studied species. We found differences concerning other nutrients: the Na mismatch for *Ch. mariana* is almost twice as high as that of the Cerambicidae beetles, whereas the Fe and Cu deficiencies for *Ch. mariana* are two to one order of magnitude lower. Thus, according to this study, different taxa of xylophagous beetles occupying the same nutritional niche may be faced with different stoichiometric mismatches concerning elements other than N and P.

### Nutritional limitations of xylophage life history and stoichiometric compensation by fungi

Walczyńska [19], [20], using experimental measurements of consumption, assimilation and growth efficiences of larvae feeding on pine wood (with stoichiometric conditions identical to the ones used in the present study), demonstrated that the low digestibility of wood may affect the life history of *S. rubra* by prolonging the development time. This result poses the following question: is the development time long enough to concentrate essential nutrients to the levels required in the body? We calculated the minimum growth period needed to collect sufficient essential element *x* (GP_x_, years) as
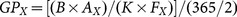
(6)where *B* – average mass of a larva, *A_X_* – concentration of element *x* in the body of an imago or pupa, *K* – daily food consumption rate averaged through the development period (after Walczyńska [19]), *F_X_* – concentration of element *x* in food. The conversion efficiency of limiting nutrients was assumed at 100%, and seasonal changes in the food consumption rate were incorporated (nothing was consumed for half the year).

Eating an exclusive diet of undecayed wood would lengthen the time needed to form body tissues to an implausible 40 years for males and 85 years for females (Table 4), and the overall assimilation efficiency would drop to improbably low values. Based on field observations, the maximum development time for *S. rubra* was estimated at three years [25]. Only highly decayed pine stumps or the material from corridors could provide the beetles with enough of the most limiting nutrients. Larvae found in highly decayed stumps were quite well developed at the age of at least 1.5 years. The stumps were likely not highly decayed during early larval development. The great majority of larvae collected from stumps were found in moderately decayed wood, which is relatively poor in nutrients (Tables 2, 4). The higher content of nutritive elements in material from corridors may suggest that larvae are capable of selecting areas of wood more heavily infected with fungi and thus providing adequate amounts of nutrients or that the activity of the larvae facilitates fungal infection through the corridors (Fig. 2). Nonetheless, the enriched chemical composition of material from corridors permits the beetles to complete their life cycle within their maximal lifetime in males (Table 4). The females must further prolong their life history to be able to assimilate the amount of nutrients sufficient to build up their bodies.

**Table 4:** Estimated minimum number of years a larva would need to spend feeding on wood of different decay categories to gather the amounts of the most limiting essential elements present in the bodies of adult beetles and pupae.

		N	P	K	Na	Cu
Male imago	Undecayed	**40**	**21**	2	1	3
	Moderately decayed	**10**	**4**	1	1	0
	Highly decayed	2	1	0	1	0
	Corridors	**3**	2	0	1	1
Female imago	Undecayed	**85**	**51**	**4**	**4**	**8**
	Moderately decayed	**21**	**10**	2	**4**	1
	Highly decayed	**4**	**3**	1	**3**	1
	Corridors	**6**	**5**	1	2	1
Pupa	Undecayed	**79**	**36**	**3**	**4**	**4**
	Moderately decayed	**19**	**7**	2	**4**	0
	Highly decayed	**3**	2	1	**3**	0
	Corridors	**6**	**4**	1	1	1

Bolded values are the estimated periods exceeding the maximum reported lifetime of *Stictoleptura rubra* larvae (3 years, see text for definitions of wood decay categories).

Wood-boring insects, enhancing the decomposition process of dead wood by mechanical grinding and fragmentation of the solid stumps, depend in turn on the fungal supplementation of their food with nutrients. Thus, elemental transport by fungi plays a pivotal role in the function of forest ecosystems: matching the stoichiometric balance between the trophic links.

## Conclusions

1. During larval development, xylophagous beetles are confronted with a severe nutritional imbalance caused by poor digestibility of food and its stoichiometric mismatch with the beetles' bodies.

2. These nutritional constraints are partly offset by the adjusted life histories of xylophages, with a development time extended to a couple of years. The life histories of dimorphic sexes and various species exploiting the same resources may differ, but computational simulations show that the prolongation of the development time is not sufficient to accumulate nutrients in adequate amounts.

3. The nutritional balance of growing xylophagous larvae can be maintained due to the substantial enrichment of dead wood with nutrients imported from the outside by the mycelium of saprotrophic fungi.

4. The fungal transfer of essential nutrients from the soil into the wood of dead trees is of fundamental importance for maintaining the detrital food web in forest ecosystems.

## Supporting Information

S1 Table **Element content in adult beetles, pupae, and wood samples from pine stumps inhabited by larvae.** Wood decay categories: 1 undecayed - hard and healthy; 2 moderately decayed – colored, moist and softer than (1) but too hard for knife; 3 highly decayed – visible changes, layers of white or brown rotting fungi, wet and soft, easily torn apart by knife or even by hand; 4 corridors – walls of corridors made by feeding larvae, together with their content.
(XLSX)

S2 Table **Dry body mass of the studied beetle species.**
(XLSX)

S3 Table **Trophic stoichiometric ratios (TSRX  =  (C:X)wood/(C:X)beetle, where C – content of carbon, X – content of element x for beetles and the potential food of their larvae.** Bolded numbers indicate the most limiting nutrients; italics – nutrients in excess. Means (white background) and confidence limits (grey background) estimated using bootstrap resampling. Wood decay categories as in Table S1.
(XLSX)

S4 Table **Ergosterol content in wood.** Wood decay categories: 1 undecayed - hard and healthy; 2 moderately decayed – colored, moist and softer than (1) but too hard for knife; 3 highly decayed – visible changes, layers of white or brown rotting fungi, wet and soft, easily torn apart by knife or even by hand.
(XLSX)
